# Supplementary material for: Impact of Dilution on Whisky Aroma: A Sensory and Volatile Composition Analysis
Source: Foods. 2023 Mar 17;12(6):1276. doi: 10.3390/foods12061276 (PMC10048241; doi:10.3390/foods12061276)
Supplement: Supplementary file 1 [file foods-12-01276-s001.zip › foods-2231799 Supplemental Materials revised.pdf]

Supplemental Materials

Table of Contents

Spirits aroma standards preparation..2

MFA groupings tables..3

Whisky names, styles, and abbreviations...10

Cochran's Q test results...11

Trained DA panel performance...12

Compounds and number codes for PLS plot...15

Correlograms of compound classes and their associated sensory attributes..19

Spirits Aroma Standard Preparation

Bacon: Put approx. 1 tsp. of crumbled bacon into each glass and cover with lid.

Source: “ends and pieces” from Market of Choice, Corvallis, OR

Preparation: Baked in drop pan at 204°C for 20 minutes and crumbled when cool.

Cedar: Put approx. 1 tbs. of cedar chips into each glass and cover with lid.

Source: https://www.amazon.com/Natural-Cedar-Chips-Mulch-Repellent/dp/B07DFPC8ZR

Cornmeal/Cooked Polenta: Put approx. 1 tbs. polenta into each glass, break up with spoon if necessary, cover with lid.

Source: San Gennaro Traditional Italian Polenta, Market of Choice, Corvallis, OR

Preparation: None

Malt: Put approx. 1 tbs. of malt into each glass and cover with lid.

Source: 6-row malt, Corvallis Brewing Supply, Corvallis, OR

Preparation: milled at purchase

Oak: Put 1 cotton ball into glass. Put 3 drops of oak extract onto cotton ball and cover with lid.

Preparation: Soak oak chips in Everclear for 72 hours.

Peat Smoke: Put approx. 1 tbs. of peated barley into each glass and cover with lid.

Source: Corvallis Brewing Supply, Corvallis, OR

Preparation: milled at purchase

Pome Fruit: Put approximately 1 tbs. of pear puree and 1 tbs. of green apple puree into each glass and cover with lid.

Source: Boiron brand green apple and Boiron brand pear puree purchased from GourmetFoodStore.com

Rubber: Put approximately 1 tbs. of rubber mulch into each glass and cover with lid.

Source: Vigoro Rubber Mulch, Home Depot, Corvallis, OR

Solvent/Chemical: Put a cotton ball into each glass and using a dropper put 4 drops of isopropyl and 4 drops of Everclear onto the cotton ball and cover with a lid.

Source: Isopropyl alcohol – Market of Choice, Corvallis, OR; Everclear – Cork and Bottle Shoppe, Corvallis, OR

Vanilla: Put a cotton ball into each glass and using a dropper put 4 drops of the vanilla extract onto the cotton ball and cover with a lid.

Source: Nielsen-Massey Madagascar Bourbon Pure Vanilla Extract, Amazon.com

Table S1. Compound classes used for creation of multi-factorial analysis (MFA) chart of 25 whiskies of various styles and brands. Data were obtained by HS-SPME-GC-MS, deconvoluted simultaneously using PARADISe deconvolution software, and tentatively identified using the NIST 2020 library. Compounds with low probability match and/or unknown source with respect to fermentation and distillation were classified as "Unknown"

Acetals, aldehydes, ketones
Ethene, ethoxy-
Acetaldehyde
Acetone
Methane, diethoxy-
Ethane, 1,1-diethoxy-
Butane, 1,1-diethoxy-3-methyl-

Acetate esters
1-Butanol, 2-methyl-, acetate
1-Butanol, 3-methyl-, acetate
Acetic acid, 2-phenylethyl ester
Lauryl acetate
Ethyl Acetate
Acetic acid, decyl ester
1-Tetradecyl acetate
Farnesol, acetate
Acetic acid, hexyl ester
Acetic acid, heptyl ester

Alcohols and fatty acids
1-Propanol
1-Propanol, 2-methyl-
1-Butanol
1-Butanol, 3-methyl-
1-Decanol
(Z)-4-Decen-1-ol
Phenylethyl Alcohol
1-Dodecanol
1-Tetradecanol
Octanoic acid
n-Decanoic acid
1-Hexadecanol
Dodecanoic acid

Phenols and aromatic hydrocarbons
o-Xylene
p-Xylene
Mesitylene
Furfural
Benzofuran

Benzaldehyde
2-Furancarboxaldehyde, 5-methyl-
Benzonitrile
Benzofuran, 2-methyl-
Naphthalene
1H-Indene, 1-ethylidene-
trans-3-Methyl-4-octanolide
Phenol, 2-methyl-
Phenol, 4-ethyl-2-methoxy-
Phenol, 3,4-dimethyl-
Phenol, 3-ethyl-5-methyl-
Phenol, 3-ethyl-
Dibenzofuran

Saturated ethyl esters
Butanoic acid, ethyl ester
Butanoic acid, 3-methyl-, ethyl ester
Pentanoic acid, ethyl ester
Hexanoic acid, ethyl ester
Heptanoic acid, ethyl ester
Octanoic acid, ethyl ester
Isopentyl hexanoate
Nonanoic acid, ethyl ester
Decanoic acid, ethyl ester
Octanoic acid, 3-methylbutyl ester
Butanedioic acid, diethyl ester
n-Capric acid isobutyl ester
Dodecanoic acid, ethyl ester
Pentadecanoic acid, 3-methylbutyl ester
Isobutyl laurate
Tetradecanoic acid, ethyl ester
Isoamyl laurate
Hexadecanoic acid, ethyl ester

Terpenes
D-Limonene
1,6,10-Dodecatrien-3-ol, 3,7,11-trimethyl-, [S-(Z)]-
o-Cymene

Unsaturated ethyl esters
8-Nonenoic acid, ethyl ester
Ethyl trans-4-decenoate
Ethyl 9-decenoate
Benzeneacetic acid, ethyl ester
10-Undecenoic acid, ethyl ester
Benzenepropanoic acid, ethyl ester
Ethyl 9-hexadecenoate
Oxalic acid, decyl 2-phenylethyl ester

Unknowns
2-Methoxy-N-methylethylamine
Pentane
Octane
Propanoic acid, 2,2-dimethyl-, octyl ester
Undecane, 2,6-dimethyl-
Toluene
1,1,6,6-Tetramethylspiro[4.4]nonane
Pentadecane
1-Decanol, 2-hexyl-
Benzo[c]thiophene
Cyclopentane, 1-ethenyl-3-ethyl-2-methyl-

Table S2. Compound classes used for the creation of multifactorial analysis charts of subset of six whiskies used for sensory analysis. Data were collected by HS-SPME-GC-MS, simultaneously deconvoluted using PARADISe software, and tentatively identified using the NIST 2020 library.

Acetals and aldehydes
Ethene, ethoxy-
Acetaldehyde
Propanal, 2-methyl-
Ethane, 1,1-diethoxy-
Hexanal
Butane, 1,1-diethoxy-3-methyl-
Benzaldehyde
2-Nonenal, (E)-
2-Propenal,
3-phenyl-
Propane, 1,1-diethoxy-2-methyl-

Acetate esters
Acetic acid, 2-methylpropyl ester
1-Butanol, 3-methyl-, acetate
Acetic acid, hexyl ester
Acetic acid, heptyl ester
Acetic acid, octyl ester
Acetic acid, nonyl ester
Acetic acid, decyl ester
5-Decen-1-ol, acetate, (E)-
Acetic acid, 2-phenylethyl ester
n-Dodecyl acetate
Ethyl Acetate

Alcohols
2-Decanol
1-Propanol
1-Propanol, 2-methyl-
1-Butanol
1-Butanol, 2-methyl-
1-Butanol, 3-methyl-
1-Decanol, 2-methyl-
1-Hexanol
1-Octanol
1-Decanol
(Z)-4-Decen-1-ol
Phenylethyl Alcohol
1-Dodecanol
1-Hexadecanol

Aromatic hydrocarbons
Styrene Benzene, 1-ethyl-3-methyl-
Benzene, 1-methyl-3-propyl-
Benzene, 1,3,5-trimethyl-
Benzene, 2-ethyl-1,4-dimethyl-
Benzene, 1-ethyl-2,4-dimethyl-
Benzene, 1,3-diethyl-5-methyl-
(1,4-Dimethylpent-2-enyl)benzene
Benzene, 1,2,3,5-tetramethyl-
Benzene, 1,2,4,5-tetramethyl-
Benzene, 4-ethenyl-1,2-dimethyl-
Benzene, 4-(2-butenyl)-1,2-dimethyl-, (E)-

Furans, benzofurans, and lactones
Furan, 2,2'-[oxybis(methylene)]bis-
Furfural
Ethanone, 1-(2-furanyl)-
Furan, 2-(1,2-diethoxyethyl)-
2-Furancarboxaldehyde, 5-methyl-
2-Furancarboxylic acid, ethyl ester
Whiskey lactone
Benzofuran
Benzofuran, 2-methyl-

Other esters
Propanoic acid, 2-methyl-, 2-methylbutyl ester
Hexanoic acid, butyl ester
Isopentyl hexanoate
n-Caprylic acid isobutyl ester
Hexanoic acid, hexyl ester
Octanoic acid, 3-methylbutyl ester
Butanedioic acid, diethyl ester
Decanoic acid, propyl ester
n-Capric acid isobutyl ester
Nonanoic acid, pentyl ester
Benzeneacetic acid, ethyl ester
Pentadecanoic acid, 3-methylbutyl ester
Benzenepropanoic acid, ethyl ester
Dodecanoic acid, propyl ester
Isoamyl laurate
Hexanoic acid, 2-phenylethyl ester
Isopropyl Palmitate
Octanoic acid, 2-phenylethyl ester

Polycyclic aromatic hydrocarbons

Naphthalene
Naphthalene, 1,2-dihydro-1,1,6-trimethyl-
Naphthalene, 1-methyl- Naphthalene, 2-ethyl-
Naphthalene, 2,6-dimethyl-
Naphthalene, 1,4-dimethyl-
Naphthalene, 2,5-dimethyl-
Naphthalene, 1,4,5-trimethyl-
Naphthalene, 2-ethenyl-
Naphthalene, 1,4,6-trimethyl-
Naphthalene, 2,3,6-trimethyl-
Dibenzofuran
Fluorene
9,10-Ethanoanthracene, 9,10-dihydro-11,12-diacetyl-
Phenols and related compounds
1,2-Dimethyl-cyclopent-2-enecarboxylic acid
Acetophenone
Phenol, 2-methoxy-4-methyl-
Phenol, 2-methyl-
Phenol, 4-ethyl-2-methoxy-
Phenol, 2,4-dimethyl-
Phenol, 3-methyl-
Phenol, 2-methoxy-4-propyl-
Phenol, 3,4-dimethyl-
Saturated ethyl esters
Formic acid, ethyl ester
Butanoic acid, ethyl ester
Butanoic acid, 2-methyl-, ethyl ester
Butanoic acid, 3-methyl-, ethyl ester
Pentanoic acid, ethyl ester
Pentanoic acid, 4-methyl-, ethyl ester
Hexanoic acid, ethyl ester
Heptanoic acid, ethyl ester
Octanoic acid, ethyl ester
Octanoic acid, 4-methyl-, ethyl ester
Nonanoic acid, ethyl ester
Decanoic acid, ethyl ester
Undecanoic acid, ethyl ester
Dodecanoic acid, ethyl ester
Tetradecanoic acid, ethyl ester
Pentadecanoic acid, ethyl ester
Hexadecanoic acid, ethyl ester
Terpenes
Limonene
o-Cymene

Longifolene
B-chamigrene
Carveol
Damascenone derivative
Calamenene
Nerolidol
Nerolidol2
Unsaturated ethyl esters
7-Octenoic acid, ethyl ester
2-Octenoic acid, ethyl ester
3-Nonenoic acid, ethyl ester
8-Nonenoic acid, ethyl ester
4-Nonenoic acid, ethyl ester
Ethyl trans-4-decenoate
Z-7-Tetradecenoic acid
Ethyl 9-hexadecenoate

Table S3. Whiskies used for volatile profiling

Whisky ***(abbreviation)***	*Style* ***(abbreviation)***
Abelour 12yr (**AB**)	Single malt Scotch (**SM**)
Basil Hayden's (**BH**)	Bourbon (**BU**)
Black Bottle (**BB**)	Blended Scotch (**BS**)
Bulleit Rye (**BU**)	Rye whiskey (**RW**)
Bunnahabhain (**BN**)	Single malt Scotch (**SM**)
Bushmill's (**BS**)	Irish whiskey (**IW**)
Dewars (**DW**)	Blended Scotch (**BS**)
Famous Grouse (**FG**)	Blended Scotch (**BS**)
Four Roses (**FR**)	Bourbon (**BU**)
George T Stagg (**GS**)	Barrel strength Bourbon (**BB**)
Glansa (**GL**)	Blended Scotch (**BS**)
Glencadam (**GC**)	Single malt Scotch (**SM**)
Highland Park (**HP**)	Single malt Scotch (**SM**)
Highland Park Dark (**HD**)	Single malt Scotch (**SM**)
Laphroaig 10yr (**LA**)	Single malt Scotch (**SM**)
Maker's Mark (**MM**)	Bourbon (**BU**)
Michter's Rye (**MR**)	Rye whiskey (**RW**)
Monkey Shoulder (**MS**)	Blended Scotch (**BS**)
Old Forester (**OF**)	Bourbon (**BU**)
Russell Reserve (**RR**)	Rye whiskey (**RW**)
Teeling (**TE**)	Irish whiskey (**IW**)
The Irishman (**IR**)	Irish whiskey (**IW**)
Tullamore Dew (**TD**)	Irish whiskey (**IW**)
Wild Turkey (**WT**)	Bourbon (**BU**)
Woodford Rye (**WR**)	Rye whiskey (**RW**)

Table S4. Cochran's Q-test for CATA results to determine sensory attributes for use with full descriptive analysis (DA) panel. Attributes in bold were chosen for DA.

Attribute	*Q*	*p-value*
**Bacon**	**40.72**	**$1.27 \cdot 10^{-2}$**
Bandaid	19.15	$6.92 \cdot 10^{-1}$
Brine	23.80	$4.14 \cdot 10^{-1}$
**Cedar**	**34.14**	**$6.31 \cdot 10^{-2}$**
**Cornmeal/Cooked polenta**	**34.33**	**$6.05 \cdot 10^{-2}$**
Hay	21.93	$5.24 \cdot 10^{-1}$
**Malt**	**29.35**	**$1.68 \cdot 10^{-1}$**
**Oak**	**42.96**	**$6.99 \cdot 10^{-3}$**
**Peat smoke**	**172.03**	**$8.66 \cdot 10^{-25}$**
Pine	16.73	$8.22 \cdot 10^{-1}$
**Pome fruit**	**76.60**	**$1.11 \cdot 10^{-7}$**
**Rubber**	**108.42**	**$4.78 \cdot 10^{-13}$**
Sawdust	26.98	$2.56 \cdot 10^{-1}$
**Solvent/Chemical**	**75.08**	**$1.95 \cdot 10^{-7}$**
**Vanilla**	**97.78**	**$3.39 \cdot 10^{-11}$**

Table S5. MANOVA F-table results of descriptive analysis (DA) trained panel performance of aroma descriptors of whisky dilution series. Statistically significant results are highlighted in bold.

	Df	*Wilks*	*F*	*p-value*
**Sample**	**23**	**0.03743**	**5.93**	**<2·10^-16^**
**Subject**	**19**	**0.00048**	**21.99**	**<2·10^-16^**
**Session**	**3**	**0.77695**	**3.02**	**1.4·10^-7^**
**Sample:Subject**	**437**	**0.00002**	**1.57**	**<2·10^-16^**
Sample:Session	69	0.15102	1.05	0.2218
**Subject:Session**	**56**	**0.06722**	**1.92**	**<2·10^-16^**

Table S6 ANOVA F-table results for all ten aroma descriptors used by descriptive analysis (DA) trained panel for whisky dilution series. Statistically significant results are highlighted in bold.

Vanilla	*Df*	*F-value*	*p-value*
**Sample**	**23**	**13.01**	**$<2\cdot10^{-16}$**
**Subject**	**19**	**55.70**	**$<2\cdot10^{-16}$**
Session	3	1.35	0.2569
**Sample:Subject**	**437**	**1.79**	**$7.95\cdot10^{-9}$**
Sample:Session	69	1.11	0.2719
**Subject:Session**	**56**	**2.03**	**$6.67\cdot10^{-5}$**

Cornmeal/Polenta	*Df*	*F-value*	*p-value*
**Sample**	**23**	**2.08**	**$2.83\cdot10^{-3}$**
**Subject**	**19**	**79.00**	**$<2\cdot10^{-16}$**
**Session**	**3**	**6.28**	**$3.68\cdot10^{-4}$**
**Sample:Subject**	**437**	**2.07**	**$1.11\cdot10^{-12}$**
Sample:Session	69	0.85	$7.87\cdot10^{-1}$
**Subject:Session**	**56**	**3.62**	**$1.10\cdot10^{-13}$**

Cedar	*Df*	*F-value*	*p-value*
Sample	23	1.38	$1.14\cdot10^{-1}$
**Subject**	**19**	**26.20**	**$<2\cdot10^{-16}$**
Session	3	1.45	$2.28\cdot10^{-1}$
Sample:Subject	437	1.03	$4.04\cdot10^{-1}$
Sample:Session	69	0.64	$9.86\cdot10^{-1}$
**Subject:Session**	**56**	**1.38**	**$4.40\cdot10^{-2}$**

Solvent/Chemical	*Df*	*F-value*	*p-value*
**Sample**	**23**	**3.35**	**$6.82\cdot10^{-7}$**
**Subject**	**19**	**41.79**	**$<2\cdot10^{-16}$**
**Session**	**3**	**3.12**	**$2.61\cdot10^{-2}$**
**Sample:Subject**	**437**	**1.87**	**$6.07\cdot10^{-10}$**
Sample:Session	69	1.08	$3.29\cdot10^{-1}$
**Subject:Session**	**56**	**2.67**	**$2.77\cdot10^{-8}$**

Malt	*Df*	*F-value*	*p-value*
Sample	23	1.46	0.081591
**Subject**	**19**	**45.32**	**$<2\cdot10^{-16}$**
**Session**	**3**	**3.15**	**$2.51\cdot10^{-2}$**
**Sample:Subject**	**437**	**1.36**	**$1.30\cdot10^{-3}$**
Sample:Session	69	0.91	$6.80\cdot10^{-1}$
**Subject:Session**	**56**	**1.53**	**$1.28\cdot10^{-2}$**

Pome fruit	*Df*	*F-value*	*p-value*
**Sample**	**23**	**9.56**	**$<2\cdot10^{-16}$**
**Subject**	**19**	**26.89**	**$<2\cdot10^{-16}$**
**Session**	**3**	**2.78**	**$4.09\cdot10^{-2}$**
**Sample:Subject**	**437**	**1.27**	**$9.31\cdot10^{-3}$**
Sample:Session	69	0.99	$5.12\cdot10^{-1}$
Subject:Session	56	1.31	$8.01\cdot10^{-2}$

Bacon	*Df*	*F-value*	*p-value*
**Sample**	**23**	**8.20**	**$<2\cdot10^{-16}$**
**Subject**	**19**	**17.15**	**$<2\cdot10^{-16}$**
Session	3	2.54	$5.60\cdot10^{-2}$
**Sample:Subject**	**437**	**1.59**	**$3.37\cdot10^{-6}$**
**Sample:Session**	**69**	**1.39**	**$2.94\cdot10^{-2}$**
**Subject:Session**	**56**	**1.71**	**$2.15\cdot10^{-3}$**

Rubber	*Df*	*F-value*	*p-value*
**Sample**	**23**	**40.66**	**$<2\cdot10^{-16}$**
**Subject**	**19**	**24.64**	**$<2\cdot10^{-16}$**
Session	3	1.34	$2.60\cdot10^{-1}$
**Sample:Subject**	**437**	**3.03**	**$<2\cdot10^{-16}$**
**Sample:Session**	**69**	**1.36**	**$4.21\cdot10^{-2}$**
**Subject:Session**	**56**	**2.36**	**$1.24\cdot10^{-6}$**

Oak	*Df*	*F-value*	*p-value*
Sample	23	1.43	$9.19 \cdot 10^{-2}$
**Subject**	**19**	**26.44**	**$<2 \cdot 10^{-16}$**
Session	3	2.23	$8.49 \cdot 10^{-2}$
**Sample:Subject**	**437**	**1.36**	**$1.20 \cdot 10^{-3}$**
Sample:Session	69	1.09	$3.02 \cdot 10^{-1}$
**Subject:Session**	**56**	**1.83**	**$5.89 \cdot 10^{-4}$**

Peat smoke	*Df*	*F-value*	*p-value*
**Sample**	**23**	**75.84**	**$<2 \cdot 10^{-16}$**
**Subject**	**19**	**18.32**	**$<2 \cdot 10^{-16}$**
**Session**	**3**	**6.87**	**$1.66 \cdot 10^{-4}$**
**Sample:Subject**	**437**	**1.87**	**$6.11 \cdot 10^{-10}$**
Sample:Session	69	0.92	$6.65 \cdot 10^{-1}$
**Subject:Session**	**56**	**1.81**	**$7.53 \cdot 10^{-4}$**

Table S7. Mixed effects model results to determine significant aroma descriptors with significant interactions

Attribute	*Interaction*	*F-value*	F_{crit} *1*	*Significance?*	F_{crit} *2*	*Significance?*
Bacon	Sample:Subject	5.17	1.554	Yes	NA	NA
Bacon	Sample:Session	5.88	NA	NA	1.687	Yes
Cornmeal/Polenta	Sample:Subject	1.00	1.554	No	NA	NA
Peat smoke	Sample:Subject	40.47	1.554	Yes	NA	NA
Pome fruit	Sample:Subject	7.52	1.554	Yes	NA	NA
Rubber	Sample:Subject	13.43	1.554	Yes	NA	NA
Rubber	Sample:Session	30.00	NA	NA	1.687	Yes
Solvent/Chemical	Sample:Subject	1.79	1.554	Yes	NA	NA
Vanilla	Sample:Subject	7.26	1.554	Yes	NA	NA

Table S8. Number codes used for PLS analysis (Figure 3) and their associated compounds.

Number Code	Compound
1	Ethene, ethoxy-
2	Acetaldehyde
3	Propanal, 2-methyl-
4	Ethane, 1,1-diethoxy-
5	Hexanal
6	Butane, 1,1-diethoxy-3-methyl-
7	Benzaldehyde
8	2-Nonenal, (E)-
9	2-Propenal, 3-phenyl-
10	Propane, 1,1-diethoxy-2-methyl-
11	Acetic acid, 2-methylpropyl ester
12	1-Butanol, 3-methyl-, acetate
13	Acetic acid, hexyl ester
14	Acetic acid, heptyl ester
15	Acetic acid, octyl ester
16	Acetic acid, nonyl ester
17	Acetic acid, decyl ester
18	5-Decen-1-ol, acetate, (E)-
19	Acetic acid, 2-phenylethyl ester
20	n-Dodecyl acetate
21	Ethyl Acetate
22	2-Decanol
23	1-Propanol
24	1-Propanol, 2-methyl-
25	1-Butanol
26	1-Butanol, 2-methyl-
27	1-Butanol, 3-methyl-
28	1-Decanol, 2-methyl-
29	1-Hexanol
30	1-Octanol
31	1-Decanol
32	Phenylethyl Alcohol
33	1-Dodecanol
34	1-Hexadecanol
35	(Z)-4-Decen-1-ol
36	Styrene
37	Benzene, 1-ethyl-3-methyl-
38	Benzene, 1-methyl-3-propyl-

39	Benzene, 1,3,5-trimethyl-
40	Benzene, 2-ethyl-1,4-dimethyl-
41	Benzene, 1-ethyl-2,4-dimethyl-
42	Benzene, 1,3-diethyl-5-methyl-
43	(1,4-Dimethylpent-2-enyl)benzene
44	Benzene, 1,2,3,5-tetramethyl-
45	Benzene, 1,2,4,5-tetramethyl-
46	Benzene, 4-ethenyl-1,2-dimethyl-
47	Benzene, 4-(2-butenyl)-1,2-dimethyl-, (E)-
48	Furan, 2,2'-[oxybis(methylene)]bis-
49	Furfural
50	Ethanone, 1-(2-furanyl)-
51	Furan, 2-(1,2-diethoxyethyl)-
52	2-Furancarboxaldehyde, 5-methyl-
53	2-Furancarboxylic acid, ethyl ester
54	Whiskey lactone
55	Benzofuran
56	Benzofuran, 2-methyl-
57	Propanoic acid, 2-methyl-, 2-methylbutyl ester
58	Hexanoic acid, butyl ester
59	Isopentyl hexanoate
60	n-Caprylic acid isobutyl ester
61	Hexanoic acid, hexyl ester
62	Octanoic acid, 3-methylbutyl ester
63	Butanedioic acid, diethyl ester
64	Decanoic acid, propyl ester
65	n-Capric acid isobutyl ester
66	Nonanoic acid, pentyl ester
67	Benzeneacetic acid, ethyl ester
68	Pentadecanoic acid, 3-methylbutyl ester
69	Benzenepropanoic acid, ethyl ester
70	Dodecanoic acid, propyl ester
71	Isoamyl laurate
72	Hexanoic acid, 2-phenylethyl ester
73	Isopropyl Palmitate
74	Octanoic acid, 2-phenylethyl ester
75	Naphthalene
76	Naphthalene, 1,2-dihydro-1,1,6-trimethyl-
77	Naphthalene, 1-methyl-
78	Naphthalene, 2-ethyl-
79	Naphthalene, 2,6-dimethyl-

80	Naphthalene, 1,4-dimethyl-
81	Naphthalene, 2,5-dimethyl-
82	Naphthalene, 1,4,5-trimethyl-
83	Naphthalene, 2-ethenyl-
84	Naphthalene, 1,4,6-trimethyl-
85	Naphthalene, 2,3,6-trimethyl-
86	Dibenzofuran
87	Fluorene
88	9,10-Ethanoanthracene, 9,10-dihydro-11,12-diacetyl-
89	1,2-Dimethyl-cyclopent-2-enecarboxylic acid
90	Acetophenone
91	Phenol, 2-methoxy-4-methyl-
92	Phenol, 2-methyl-
93	Phenol, 4-ethyl-2-methoxy-
94	Phenol, 2,4-dimethyl-
95	Phenol, 3-methyl-
96	Phenol, 2-methoxy-4-propyl-
97	Phenol, 3,4-dimethyl-
98	Formic acid, ethyl ester
99	Butanoic acid, ethyl ester
100	Butanoic acid, 2-methyl-, ethyl ester
101	Butanoic acid, 3-methyl-, ethyl ester
102	Pentanoic acid, ethyl ester
103	Pentanoic acid, 4-methyl-, ethyl ester
104	Hexanoic acid, ethyl ester
105	Heptanoic acid, ethyl ester
106	Octanoic acid, ethyl ester
107	Octanoic acid, 4-methyl-, ethyl ester
108	Nonanoic acid, ethyl ester
109	Decanoic acid, ethyl ester
110	Undecanoic acid, ethyl ester
111	Dodecanoic acid, ethyl ester
112	Tetradecanoic acid, ethyl ester
113	Pentadecanoic acid, ethyl ester
114	Hexadecanoic acid, ethyl ester
115	Limonene
116	o-Cymene
117	Longifolene
118	B-chamigrene
119	Carveol
120	Damascenone derivative

121	Calamenene
122	Nerolidol
123	Nerolidol2
124	7-Octenoic acid, ethyl ester
125	2-Octenoic acid, ethyl ester
126	3-Nonenoic acid, ethyl ester
127	8-Nonenoic acid, ethyl ester
128	4-Nonenoic acid, ethyl ester
129	Ethyl trans-4-decenoate
130	Z-7-Tetradecenoic acid
131	Ethyl 9-hexadecenoate

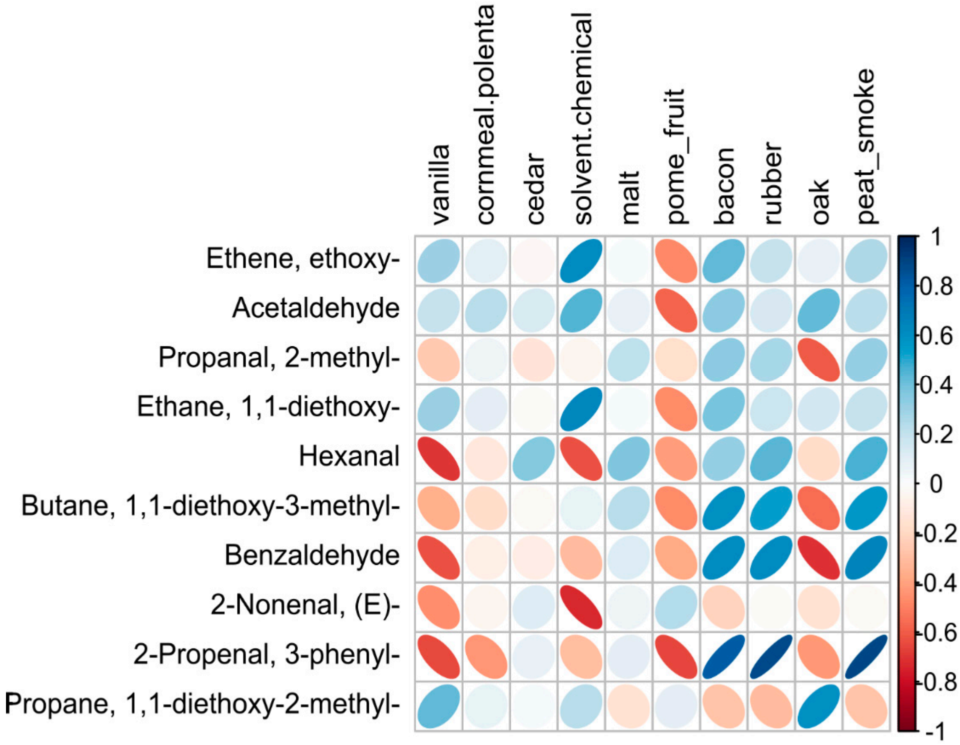

Figure S1. Correlogram of "acetal and aldehyde" compound class and sensory attributes of six whiskies (three Bourbons three Scotches)

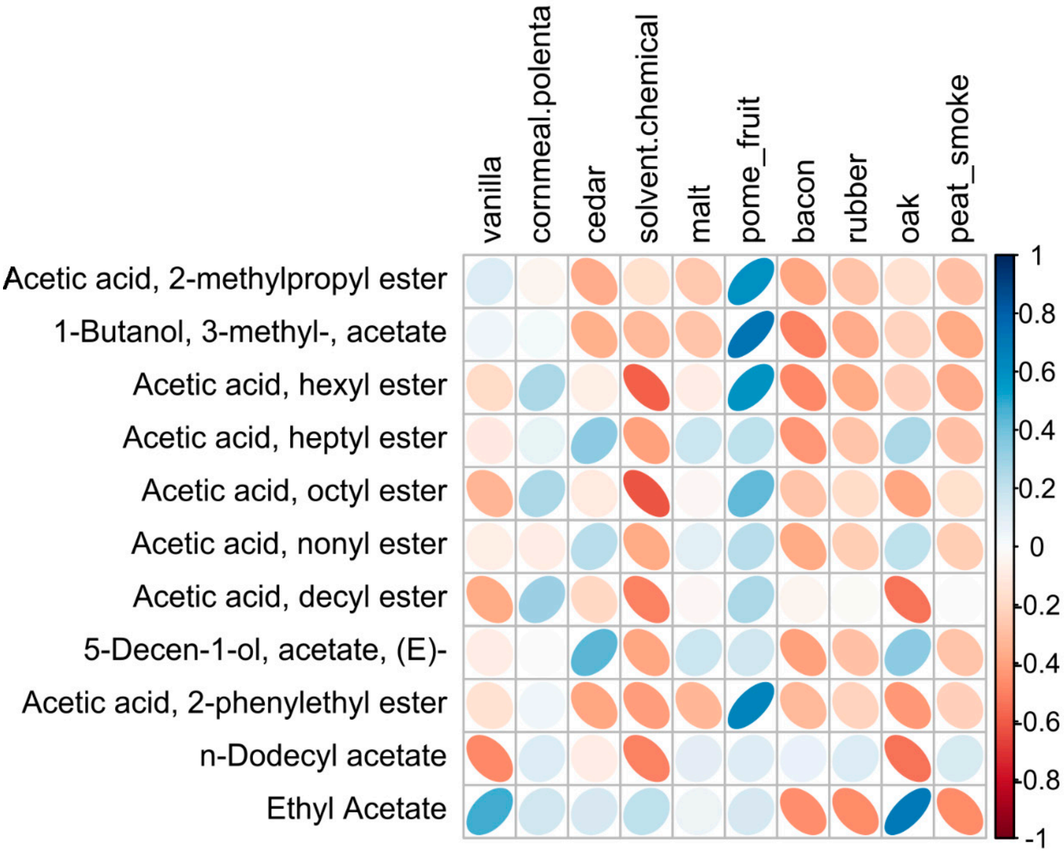

Figure S2. Correlogram of "acetate ester" class of compounds and sensory attributes of six whiskies.

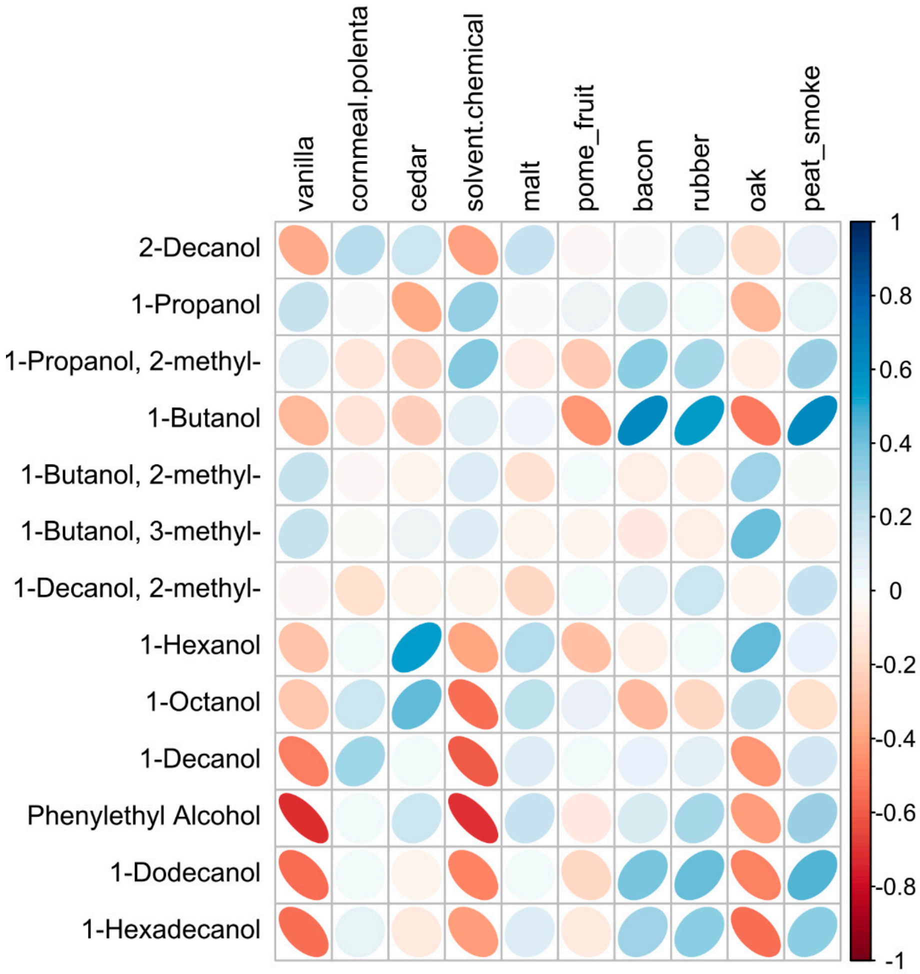

Figure S3. Correlogram of "alcohols" compound class and sensory attributes of six whiskies.

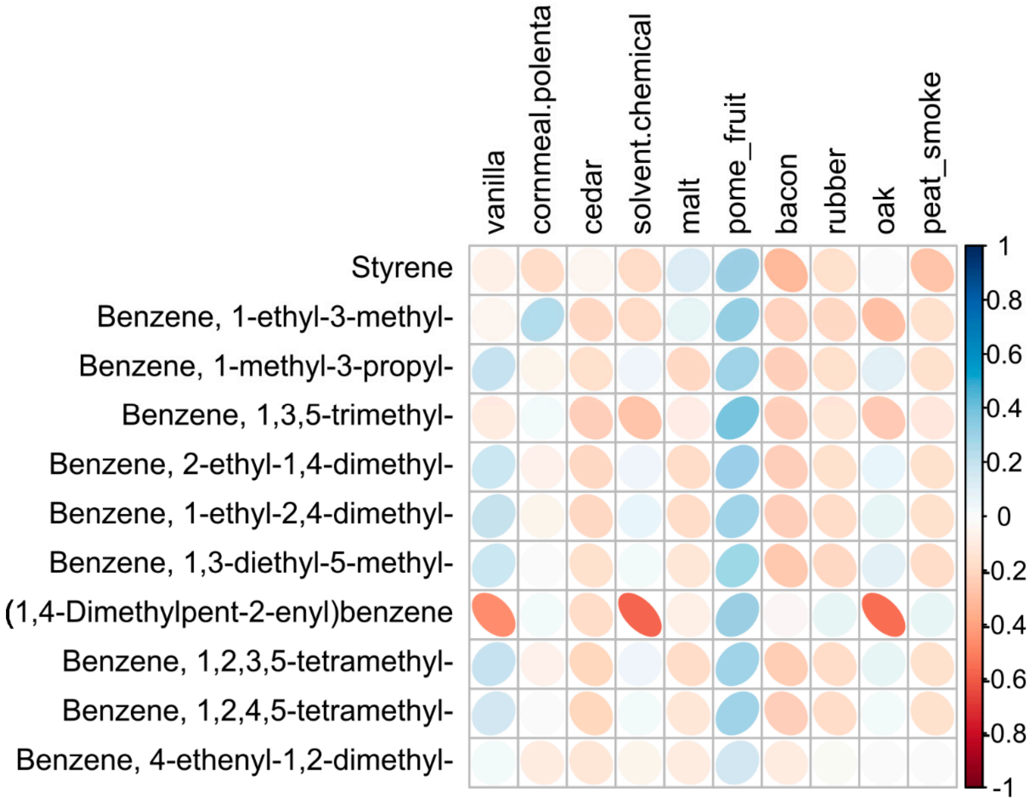

Figure S4. Correlogram of "aromatic hydrocarbon" compound class and sensory attributes.

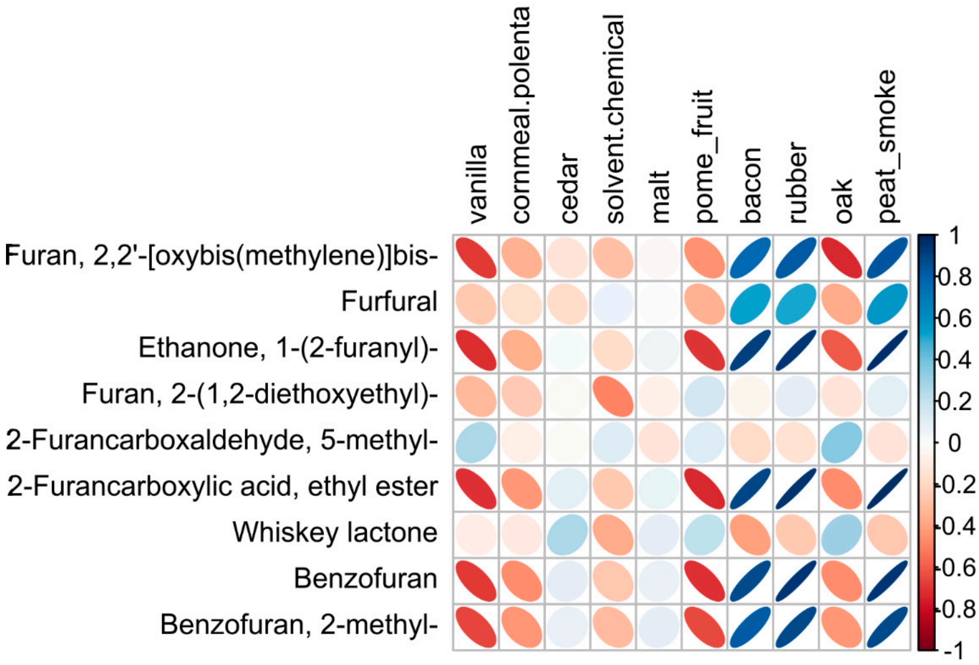

Figure S5. Correlogram of "furan and benzofuran" class of compounds and sensory attributes.

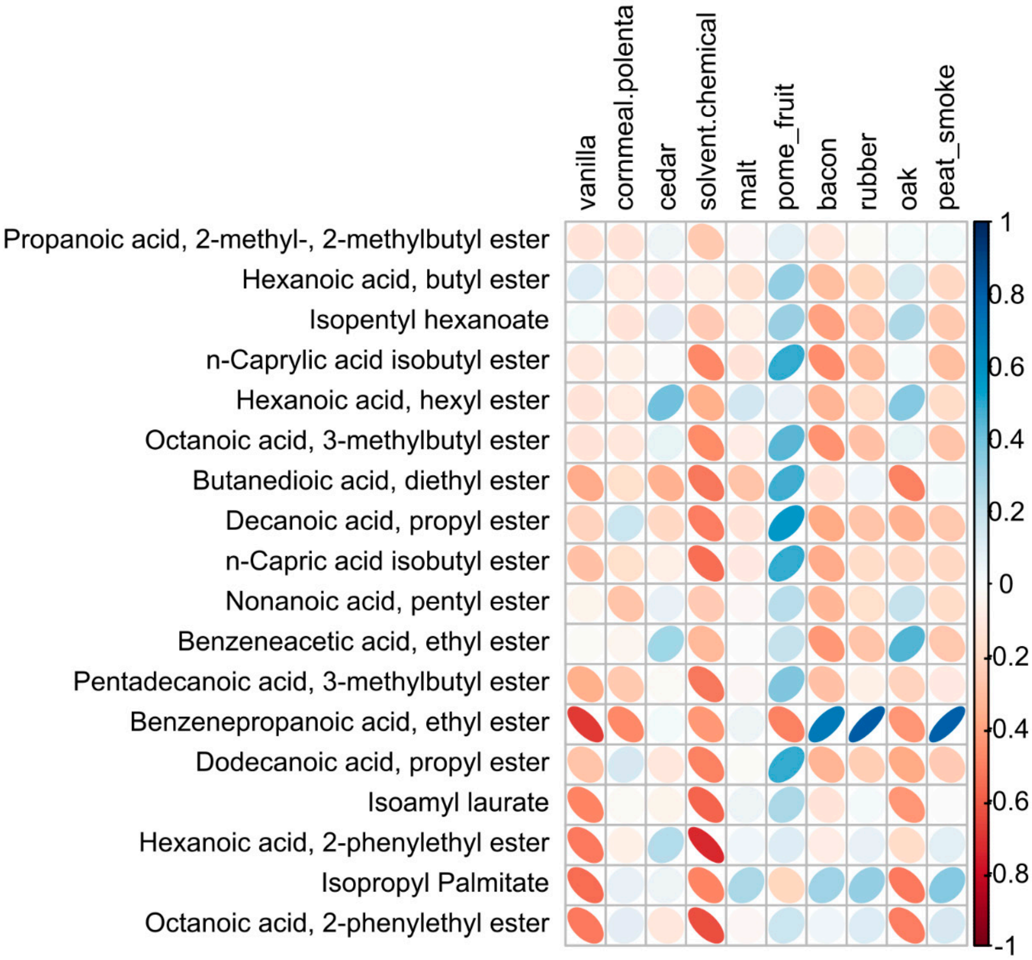

Figure S6. Correlogram of “other esters” class of compounds and sensory attributes.

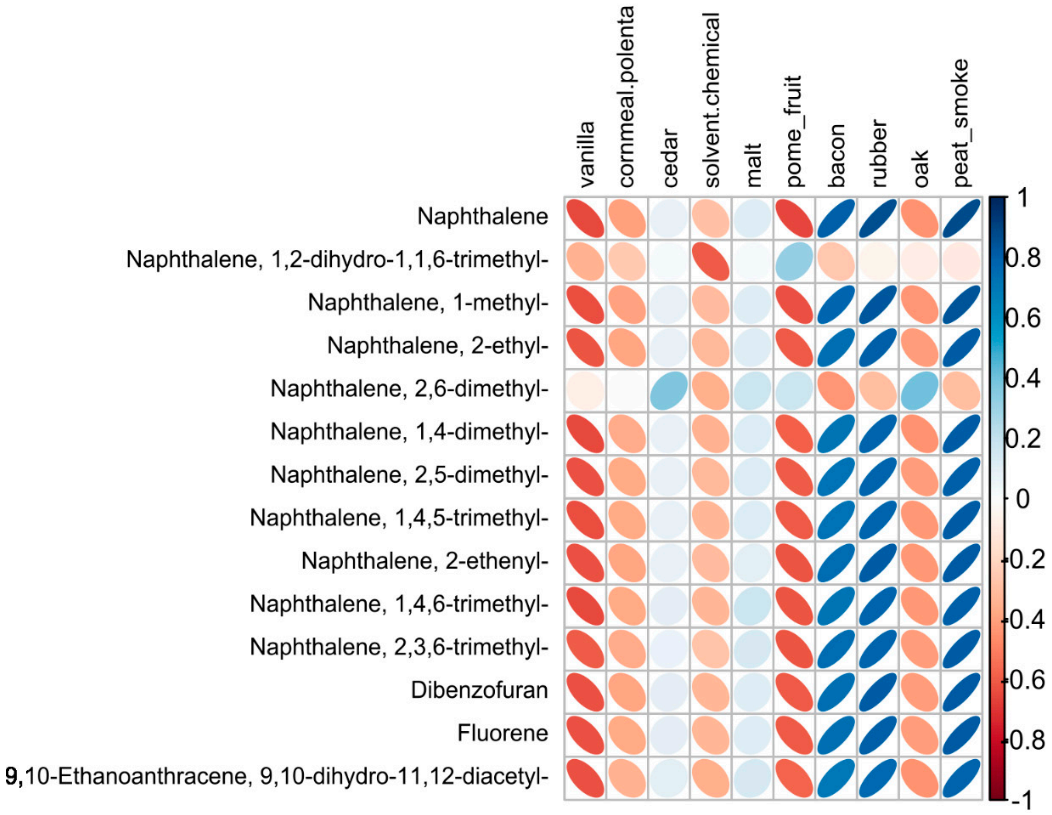

Figure S7. Correlogram of “polycyclic aromatic hydrocarbon” class of compounds and sensory attributes.

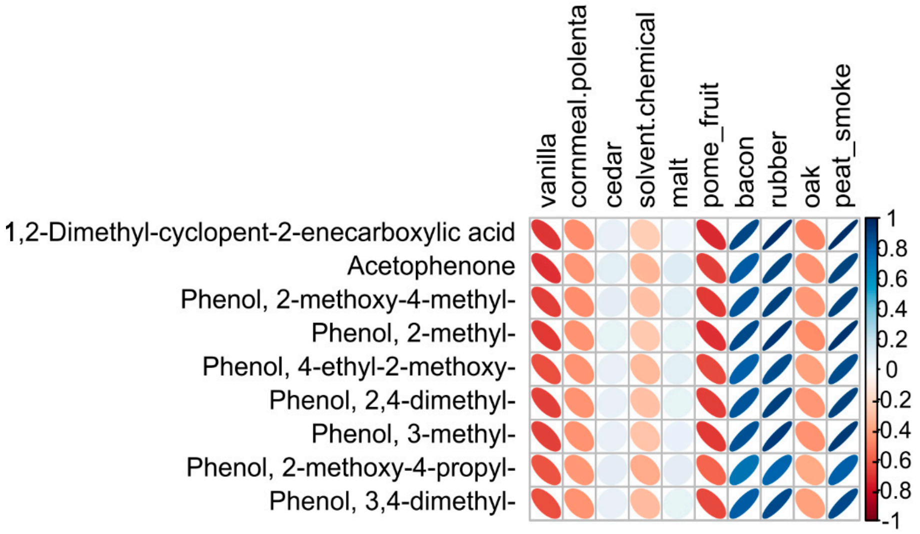

Figure S8. Correlogram of "phenol" compound class and sensory attributes.

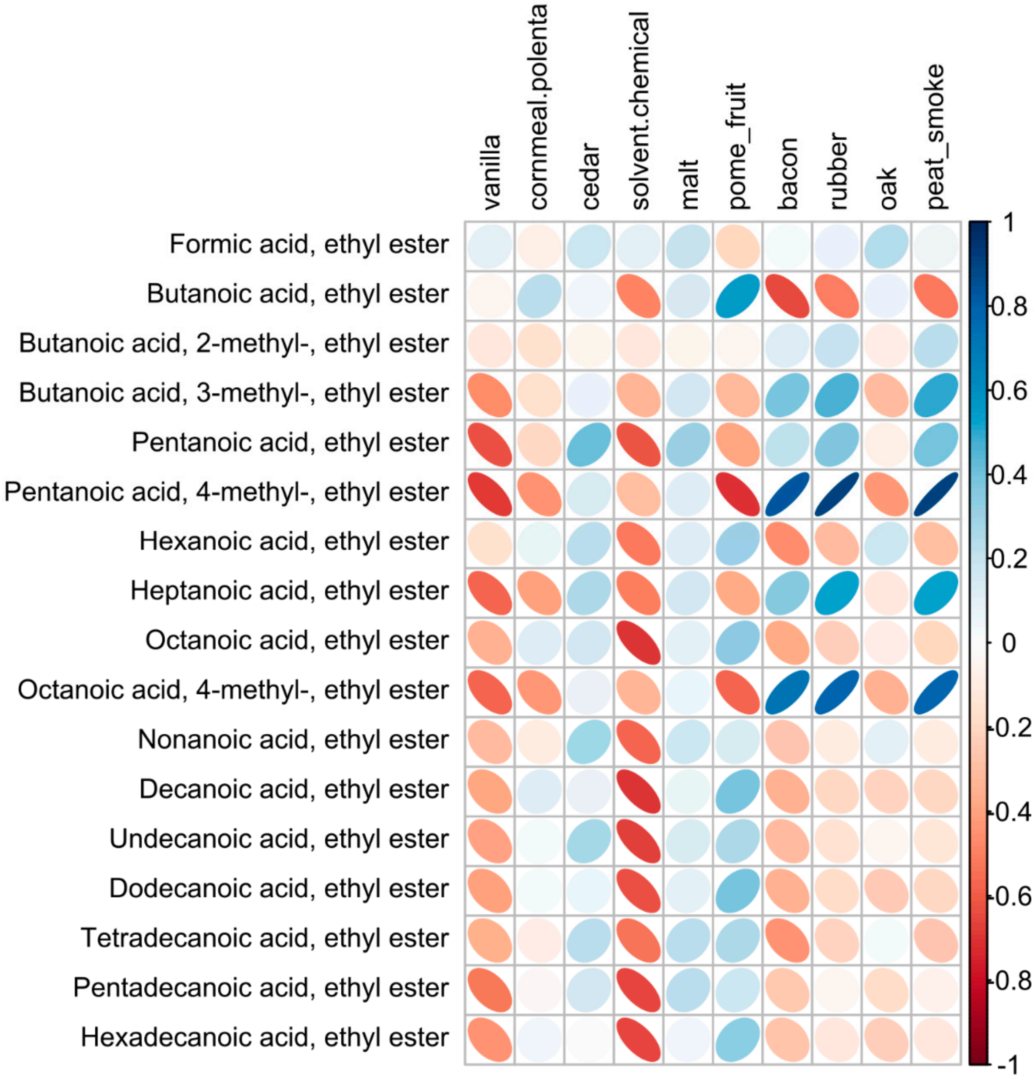

Figure S9. Correlogram of "saturated ethyl ester" compound class and sensory attributes.

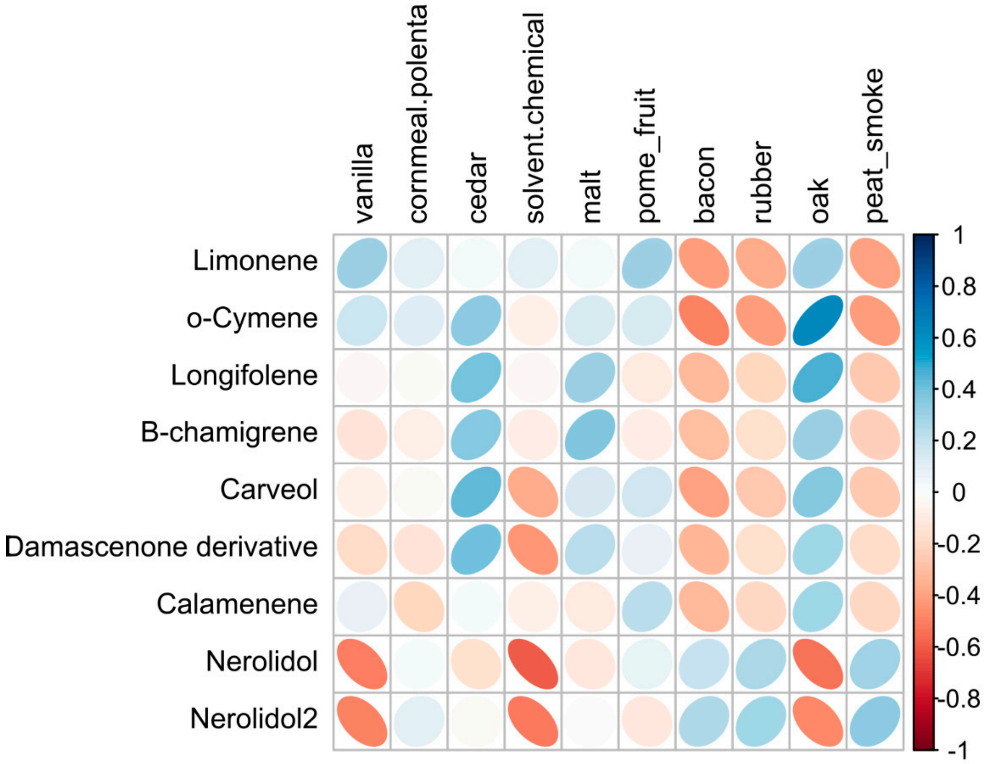

Figure S10. Correlogram of "terpenes" compound class and sensory attributes

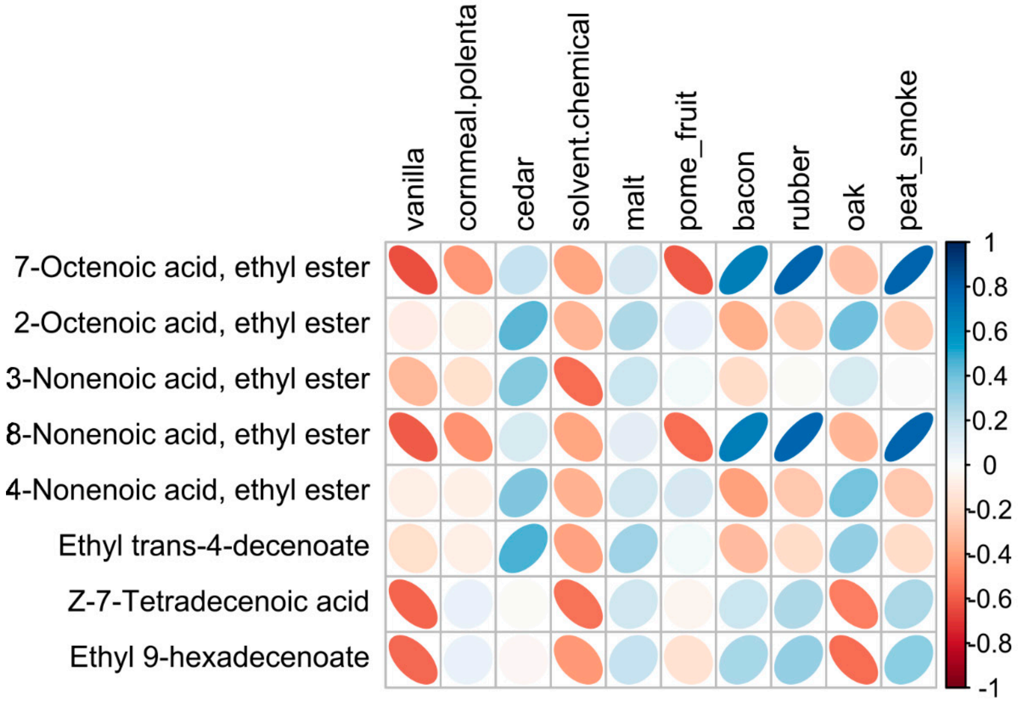

Figure S11. Correlogram of "unsaturated ethyl ester" compound class and sensory attributes.
